# Antioxidative and Potentially Anti-inflammatory Activity of Phenolics from Lovage Leaves *Levisticum officinale* Koch Elicited with Jasmonic Acid and Yeast Extract

**DOI:** 10.3390/molecules24071441

**Published:** 2019-04-11

**Authors:** Urszula Złotek, Urszula Szymanowska, Łukasz Pecio, Solomiia Kozachok, Anna Jakubczyk

**Affiliations:** 1 Department of Biochemistry and Food Chemistry, University of Life Sciences in Lublin, Skromna Str. 8, 20-704 Lublin, Poland; urszula.szymanowska@up.lublin.pl; 2 Department of Biochemistry and Crop Quality, Institute of Soil Science and Plant Cultivation—State Research Institute, Czartoryskich 8, 24-100 Puławy, Poland; lpecio@iung.pulawy.pl (Ł.P.); skozachok@iung.pulawy.pl (S.K.)

**Keywords:** lovage, elicitation, phenolic compounds, antioxidant activities, anti-inflammatory properties

## Abstract

The effect of elicitation with jasmonic acids (JA) and yeast extract (YE) on the production of phenolic compounds as well as the antioxidant and anti-inflammatory properties of phenolic extracts of lovage was evaluated. The analysis of phenolic compounds carried out with the UPLC-MS technique indicated that rutin was the dominant flavonoid, while 5-caffeoylquinic acid was the main component in the phenolic acid fraction in the lovage leaves. The application of 10 µM JA increased the content of most of the identified phenolic compounds. The highest antioxidant activities estimated as free radical scavenging activity against ABTS (2,2′-azino-bis(3-ethylbenzothiazoline-6-sulphonic acid) and reducing power were determined for the sample elicited with 10 µM JA, while this value determined as iron chelating ability was the highest for the 0.1% YE-elicited lovage. The 0.1% and 1% YE elicitation also caused significant elevation of the lipoxygenase (LOX) inhibition ability, while all the concentrations of the tested elicitors significantly improved the ability to inhibit cyclooxygenase 2 (COX2) (best results were detected for the 10 µM JA and 0.1% YE2 sample). Thus, 0.1% yeast extract and 10 µM jasmonic acid proved to be most effective in elevation of the biological activity of lovage.

## 1. Introduction

Lovage (*Levisticum officinale* Koch.) belongs to the *Apiaceae* family. *Apiaceae*, which is one of the largest plant families, comprises many culinary and medicinal plants usually characterized by a pungent or aromatic smell due to the presence of essential oil. Additionally, herbs from the *Apiaceae* family possess many compounds exerting different biological effects: they possess antioxidant, antibacterial, hepatoprotective, vaso-relaxant, cyclooxygenase inhibitory, and antitumor activities [1]. In many areas, natural wild plant communities of some rare species such as *L. officinale* have been threatened; therefore, domestic cultivation of these plants may be an alternative way to produce valuable herbs material [2]. One of the herb-derived groups of compounds raising great scientists’ interest is polyphenols, as they can help protect the human organism against oxidative stress and inflammatory processes. Recently, there has been growing interest in the high dietary content of phenolic compounds due to their multiple biological activities related to the prevention of many diseases such as cardiovascular diseases, cancer, diabetes, and Alzheimer’s [3,4,5].

For this reason, there is still interest in the search for new methodologies to increase the polyphenol content in edible plants. It is well known that the content of bioactive compounds in plants depends on many factors, especially genetic (family, species, cultivar, etc.), physiological (organ, maturity, and age), and environmental (fertilization, stress, or some treatments like elicitation). As a method of inducing plant secondary metabolism, elicitation can be proposed as a new, non-conventional, and ecologically-friendly approach for plant protection as well as treatment enhancing the synthesis of phytochemicals [6]. Plant response to the elicitor treatment depends on the physiological condition of plants and on factors related to elicitation e.g. the dose, nature, way of elicitor application, and treatment time [6]. Based on their nature, the elicitors can be either abiotic and biotic. There are many examples of abiotic chemical elicitors in the literature which influence the biosynthesis of secondary metabolites by plants, such as inorganic salts and metal ions [6] as well as plant hormones (arachidonic acid or jasmonic acid) [7,8,9]. Jasmonic acid, which is a lipid-derived plant compound and a plant phytohormone, also acts as an elicitor inducing secondary metabolism of many plants when applied exogenously [10,11]. Complex biological preparations such as yeast extract and microbial cell-wall preparations have often been used as biotic elicitors in in vitro plant cultures [12]. Jasmonic acid is an abiotic elicitor: it is exactly one compound while some biotic elicitors like yeast extract are elicitors where the molecular structure of active compound is not fully known, but there are many studies confirm that they can act as elicitor. Yeast extract is rich in vitamin B-complex and contents essential components like chitin, *N*-acetyl-glucosamine oligomers, β-glucan, glycopeptides, and ergosterol. Biotic elicitors like yeast extract can substitute for fungal elicitors during a pathogen attack [12,13,14]. There are only few examples of yeast extract used to enhance the production of phytochemicals in whole plants [15,16].

The aim of this study was to evaluate the influence of jasmonic acid and yeast extract elicitation on the content and the antioxidant and potentially anti-inflammatory activity of phenolic compounds in *L. officinale* (Koch). An additional objective of the present research was to select the best concentrations of the elicitors used to enhance lovage health-promoting qualities.

## 2. Results

Table 1 presents the qualitative and quantitative analysis of phenolic compounds in the leaves of the control and elicited lovage conducted with the UPLC-MS technique.

The sum of phenolic compounds in the leaves of the control plants was 48.43 ± 4.02 mg/g dw. In lovage treated with 10 µM JA, the content of total phenolic compounds was significantly higher (by 55% in comparison to the control). Using the UPLC-MS technique, 14 compounds were identified in the tested samples and elicitation did not cause any qualitative change, while some quantitative changes in were observed (Table 1). In all samples, 5-caffeoylquinic acid was the dominant phenolic acid derivative (the content between 6.01 and 19.12 mg/g dw), whereas rutin constituted the largest flavonoid fraction (the content ranged from 9.85 mg/g dw to 18.60 mg/g dw). Additionally, a compound from the group of furanocoumarins (i.e., apterin) was identified as one of the dominant compounds in the lovage leaves (Table 1). It should be noted that the elicitation with JA2 resulted in an increase in the content of many of phenolic compounds in lovage leaves; the largest increase in the content was recorded in the case of 5-caffeoylquinic acid (increase by 89.49%) (Table 1). Very important results were also obtained in the case of a compound from the group of phthalides (i.e., ligustilide), as the elicitation with both JA and YE (all tested concentrations of elicitors) resulted in a very large increase in its content in the lovage leaves (Table 1). In the case of jasmonic acid, the best result was obtained after using 10 µM (JA2) of the elicitor (about a threefold increase in the ligustilide content), while the most effective concentration of the yeast extract was 0.1% (YE2), which yielded an approximately 1.7-fold increase in the ligustilide content (Table 1). Summarizing the elicitation with jasmonic acid (JA2) resulted in a significant increase in the sum of phenolic compounds and the majority of identified compounds, while elicitation with yeast extract resulted in a statistically significant increase in ligustilide content only (the highest in the case of using YE2 (Table 1).

**Figure 1:**
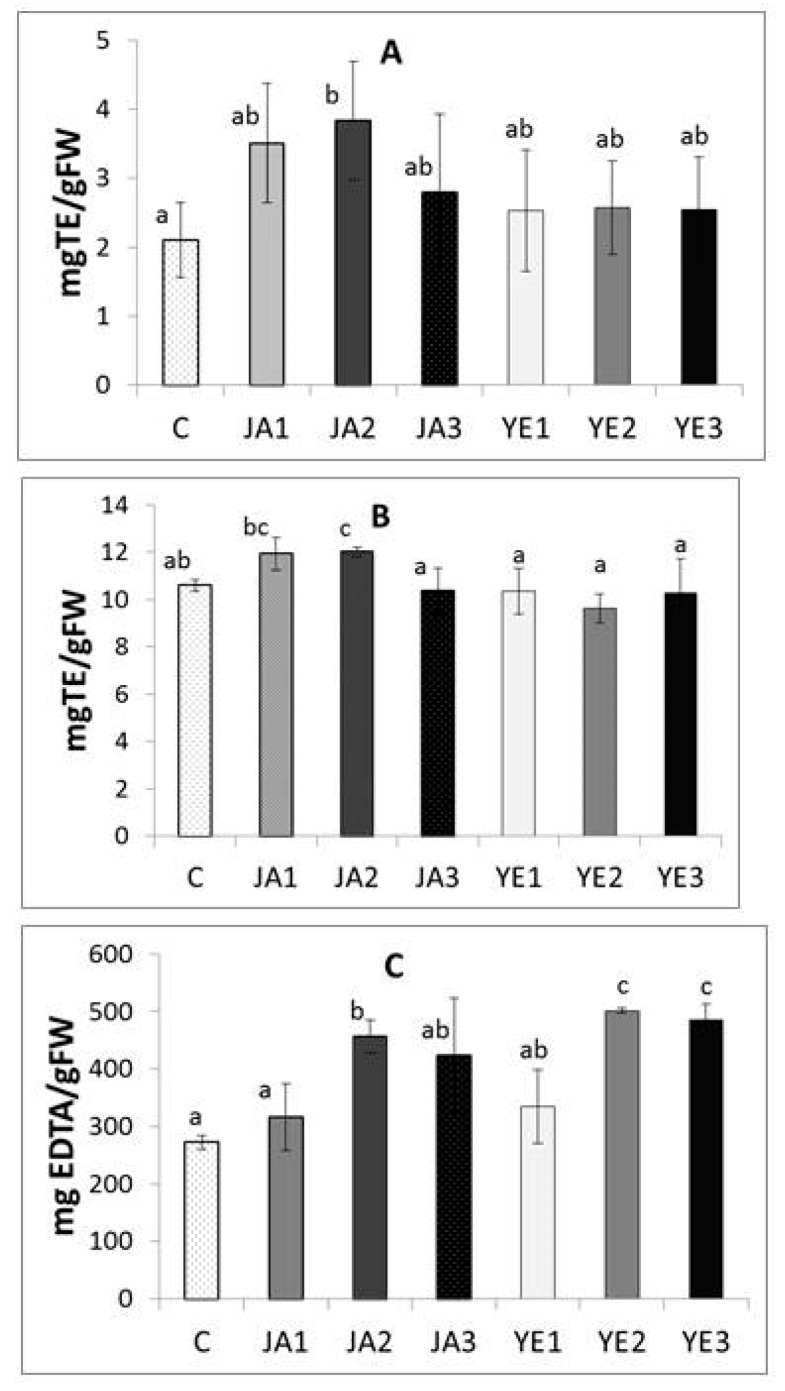
Effect of elicitation on antioxidant activity of phenolic extracts from lovage: antiradical activity (**A**), reducing power (**B**), chelating power (**C**); C–control plants, JA1–plants elicited with 1 µM jasmonic acid, JA2–plants elicited with 10 µM jasmonic acid JA3–plants elicited with 100 µM jasmonic acid; YE1–plants elicited with 0.01% yeast extract, YE2–plants elicited with 0.1% yeast extract, YE3–plants elicited with 1% yeast extract. Results are means ± SD of three independent measurements. Different letters indicate significantly differences (*p* < 0.05).

The antioxidant activities of the extract from fresh lovage leaves (control and elicited) were determined using three complementary methods: free radical scavenging activity against ABTS, reducing power, and iron chelating activity (Figure 1).

A statistically significant increase in the ability to neutralize ABTS radical and reducing power in the studied extracts was observed only after the JA2 elicitation (an increase by 80.95% and 13.3% in comparison to the control, respectively), as seen in Figure 1. The JA2 sample was also characterized by a high iron chelating ability (456.43 mg EDTA/g FW vs. 272.54 mg EDTA/g FW for the control sample), but it should be noted that the highest iron-chelating abilities were detected for the YE2 and YE3 samples (a 1.84-fold and 1.78-fold increase, compared to the control, respectively) (Figure 1).

**Figure 2:**
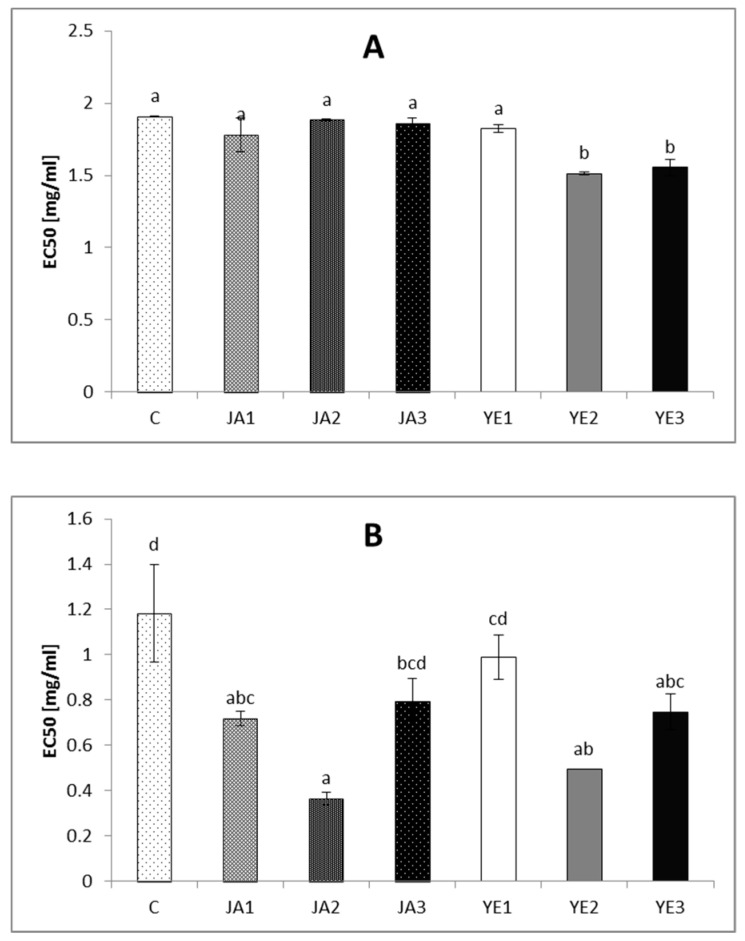
Lipoxygenase (**A**) and cyclooxygenase (**B**) inhibition by phenolics from lovage induced by elicitors; C–control plants, JA1–plants elicited with 1 µM jasmonic acid, JA2–plants elicited with 10 µM jasmonic acid JA3–plants elicited with 100 µM jasmonic acid; YE1–plants elicited with 0.01% yeast extract, YE2–plants elicited with 0.1% yeast extract, YE3–plants elicited with 1% yeast extract. Results are means ± SD of three independent measurements. Different letters indicate significantly differences (*p* < 0.05).

As shown in Figure 2, the extract from the studied lovage leaves also exhibits the ability to inhibit pro-oxidative enzymes such as lipoxygenase and cyclooxygenase 2.

The highest LOXI activity was detected for YE2 and YE3 (the EC50 values were 20.52% and 18.42% lower, respectively, than those in the control sample) (Figure 2). Surprisingly, the elicitation with JA did not influence the ability to inhibit LOX by the extract from lovage leaves. In turn, all the concentrations of the tested elicitors significantly influenced the ability to inhibit COX2 by the extracts from lovage leaves (Figure 2). The best results were obtained after using JA2 and YE2 for the elicitation: there was an increase in the COX2 inhibition potential by 69.49% and 58.47% in relation to the control, respectively.

## 3. Discussion

Although there are many examples of cell culture-based elicitation, considerably less research has been conducted in whole plant systems and elicitation is still underutilized in the production of medicinal plants [17,18,19]. Additionally, the effect of cell culture elicitation is difficult to transfer directly to experiments with whole plants, because there are numerous factors that may be specific to the response of whole plants, for example plant signal transport or the physiological and developmental stage of the plant [13]. In investigations of whole plants, it should also be taken into account that abiotic and biotic elicitors act similarly to stress factors [13]. The pathways responsible for acquiring systemic immunity are activated in the plant, but the same metabolic pathways increase the production of many secondary metabolites [13]. However, the stress level (and therefore the concentration of elicitors) is also important, because there are some studies indicating that too high elicitor concentrations resulted in a decrease in the secondary metabolite content. For example, in a study conducted by Kuzel et al. [20], foliar spray with 10 µM salicylic acid was more efficient for the induction of the phenolic content in tops of purple coneflower (*E. purpurea* L. Moench.) than 100 µM salicylic acid. Similarly, the investigations conducted by Złotek et al. [7] indicated that 1 µM jasmonic acid was more effective in enhancement of production of phenolic compounds in lettuce leaves than 100 µM jasmonic acid. In the present study, two biotic [YE] and abiotic (JA) elicitors (each in three concentrations) were used for elicitation of lovage. Only the elicitation with JA2 resulted in a statistically significant increase in the sum of phenolic compounds (Table 1). It should also be noted that the YE2 sample (elicited with 0.1% YE) was characterized by the highest content of phenolic compounds (Table 1). As indicated in other studies, the use of jasmonic acid resulted in increased production of secondary plant metabolites (including phenolic compounds), as well as in other plants, such as basil [11,21,22] or lettuce [7]. The other abiotic elicitor (salicylic acid) caused an increase in the total phenolic content in lovage as well [23]. Yeast extract was used as a biotic elicitor, especially in in vitro cultures of medicinal plants, as seen in previous research [19,24]. Overproduction of phenolic compounds by YE and salicylic acid elicitation was observed in cultured ginger tissues [19]. A majority of research on the use of yeast extract as a biotic elicitor reported in the literature was conducted in suspension cell cultures, and only a few reports present the effect of YE application to plants in vivo. In the research conducted by Złotek [16], elicitation with YE, as in the present study, did not increase the content of phenolic compounds but significantly increased the content of ascorbic acid and chlorophyll in marjoram leaves. Additionally, another biotic elicitor, chitosan oligosaccharide, was an effective stimulator of polyphenol production by oregano plants [25]. Because the profile of phenolic compounds is equally important for biological activity [26], the qualitative and quantitative analysis of phenolic compounds in the studied plants was carried out with the use of the UPLC-MS technique. This analysis indicated that rutin, quercetin, and kaempferol derivatives were identified in the flavonoid fraction in studied lovage leaves, with rutin as the dominant flavonoid. In turn, caffeic acid derivatives were dominant in the phenolic acid fraction (Table 1). These results partially agree with previous literature data based on HPLC analysis indicating rutin and quercetin as flavonoids present in lovage leaves, although myricetin was also identified in this research [3]. On the other hand, in the research reported by Justesen and Knuthsen [27] (HPLC-DAD technique—High-Performance Liquid Chromatography with Diode-Array Detection), only quercetin and kaempferol were identified in lovage leaves. Synapic acid predominated among the phenolic acids and their derivatives in the work cited above [3], while the highest amounts of caffeic acid derivatives (among which 5-caffeoylquinic acid was predominant) were determined in the lovage analyzed in our study (Table 1). Similarly, in the studies conducted by Justesen [28], in which the identification was made using the LC-MS technique, 3-caffeoylquinic acid was identified in the phenolic acid fraction in lovage leaves. However, in the case of flavonoids, the results in the present study did not completely confirm research mentioned above, because luteolin and quercetin rhamnoglucosides were detected in the lovage leaves in the latter work [28]. These differences may result from the difference in the lovage variety used for the research, the method of phenolic compound extraction, and the techniques used for identification thereof. The elicitation used in the present study did not cause qualitative changes in the examined phenolic compounds, but it should be noted that an increase in the content of many identified phenolics was observed in the plants treated with JA2 (Table 1). In the context of quality of elicited plants, as previous research has indicated, high content of furanocoumarin can provide bitterness. In the present study, elicitation with JA2 resulted in a slight increase of compounds in the group of furanocoumarin (apterin) content in lovage leaves, but it should be noted that it was not a statistically significant change. Similarly, in a research conducted by Heredia and Cisneros [29], elicited with methyl jasmonate carrot, isocoumarin levels accumulated were minimal, but isocoumarin content in carrot was mostly affected by the ethylene treatment. However, because the content of some apterin derivatives (like caffeic acid and apterin ester and sinapic acid and apterin ester) increased in the elicited with JA lovage leaves in our studies, further research in the context of the quality of the elicited herbs should be continued.

It is well known that phenolic compounds possess many biological activities. The pro-health impact of polyphenols is related mainly to their commonly reported antioxidant properties resulting from their ability to neutralize free radicals, disrupt autooxidation chain reactions, chelate transition metal ions, and inhibit the activity of pro-oxidant enzymes (e.g., lipoxygenase or cyclooxygenase) [30,31].

Lovage is a culinary and medicinal herb characterized by high levels of phenolic compounds with documented antioxidant properties [3]. The present study confirms this observation, but the novelty of the present research is the use of jasmonic acid and yeast extract elicitation in the production of lovage in order to improve its biological properties. As indicated in our study, the antiradical and reducing potential of lovage extracts was evaluated mostly by JA elicitation (especially JA2), while the ability to chelate iron ions increased the most after the YE2 elicitation (Figure 1). Similarly, in studies on marjoram and basil elicitation, the use of jasmonic acid resulted in an increase in antiradical properties (marjoram and basil) and reducing power (marjoram) of this herb [16,21], as well as improvement of DPPH-free radical scavenging activity of the sweet basil after 0.1 and 0.5 mM methyl jasmonate treatments [8].

Inflammation plays an important role in various diseases, such as rheumatoid arthritis, atherosclerosis, and asthma, which still constitute the main health problems of the world’s population. Many medicinal plants have the ability to synthesize a variety of secondary metabolites with anti-inflammatory activity, such as ingredients of essential oil or phenolic compounds. The use of herbal medicines is becoming popular due to its safety and lack of toxicity compared to traditional medicines [32]. As indicated in many studies, the potential anti-inflammatory activities of herbal plant compounds are mainly related to their inhibition of arachidonic acid metabolism, especially via inhibition of pro-oxidative enzymes (e.g., cyclooxygenase (COX) and lipooxygenase (LOX)) [33,34]. In the present study, the phenolic compounds from the lovage leaves showed LOX and COX 2-inhibitory potential, but it should be noted that elicitation with all the tested concentrations of inducers significantly increased the inhibition of cyclooxygenase 2 (the JA2 and YE2 variants were the most effective) (Figure 2). Additionally, YE elicitation (YE2 and YE3) caused an increase in the ability to inhibit LOX by the studied extracts (Figure 2). The mechanism of potential inflammatory mechanism of action LOX and COX is associated with biosynthesis of inflammatory mediators like eicosanoids. Eicosanoids are associated with inflammatory disorders, but it should be emphasized that among the eicosanoid generating enzymes, COX2 was to be essential for production of prostaglandins in inflammatory sites, while COX1 plays a significant role in physiological processes [35]. Additionally, lipoxygenases (LOXs) are a family of non-heme iron-containing dioxygenases catalyzing the biosynthesis of leukotrienes. Leukotrienes function as initiators of inflammation and their inhibition is considered to be partly responsible for the anti-inflammatory activity. Therefore, many studies are looking for natural compounds that act as dual COX-2/5-LOX inhibitors as potentially anti-infammatory agents [36,37]. Many investigations have indicated that bioactive compounds from some herbs possess anti-inflammatory potential due to inhibition of LOX and COX enzymes, but only a few reports present the effect of elicitation on these activities of plant phytochemicals. As in the present study, in research conducted by Złotek et al. [21], elicitation with jasmonic acid increased the ability of a purple basil phenolic-rich fraction to inhibit COX activity but did not improve the LOX inhibition ability. In turn, the anthocyanin fraction from jasmonic acid-induced basil showed a lower ability to inhibit COX activity, compared to the control [9]. It should be also noted that the elevation of the LOX inhibitory potential caused by the yeast extract elicitation noted in this study corresponds with the results obtained by Złotek and Świeca [15], which indicate that elicitation with yeast extract caused an increase in the ability to inhibit LOX activity by phenolics from lettuce.

Some literature data indicated that such solvents as methanol or ethanol can be used also for extraction of phthalide compounds from medicinal herbs [38]. In our study, one of the phthalide compounds (i.e., ligustilide) was detected in the methanolic extract of the control and elicited lovage (Table 1). Ligustilide is often the major phthalide isolated from *Apiaceae* herbs, to which many biological activities—including anti-inflammatory, anti-oxidative, antibacterial, antifungal, antiviral activities—modulate the central nervous system and cardiac function, while others are assigned [38,39]. In the present study, the elicitation with JA and YE resulted in a significant increase in the content of this compound in the lovage leaves (Table 1), which may have contributed to the improved biological activity of the elicited herbs.

## 4. Materials and Methods 

### 4.1. Plant Materials and Growth Conditions

Lovage seeds (*Levisticum officinale* Koch. cv. Elsbetha) were derived from Enza Zaden Company, Enkhuizen, The Nethherlands. The plants were grown in a growth chamber (SANYO MLR-350H) at 25/18 °C, with a photoperiod 16/8 h day/night, a photosynthetic photon flux density (PPFD) at a plant level of 500–700 µmol m^−2^s^−1^, and a relative humidity of 75%. The herb seeds were sown into sowing boxes filled with universal soil for sowing seeds. Seven-day-old seedlings were transplanted to 600 mL pots containing universal garden soil (four plants per pot). The plants were watered every other day and fertilized twice—for the first time before plant transplanting and the second time one week after transplanting. Twenty-one day-old plants were sprayed with a water solution of the tested elicitors (1.5 mL per plant): 1 µM jasmonic acid (JA1), 10 µM jasmonic acid (JA2), 100 µM jasmonic acid (JA3), 0.01% yeast extract (YE1), 0.1% yeast extract (YE2), and 1% yeast extract (YE3). 0.01% Tween 20 was used as a surfactant. The concentrations of elicitors used in this work, as well as growing and elicitation conditions were selected based on previous screening experiments (data not published), so as not to induce negative effects on the health and growth of plants and based on literature [7,15]. Twenty-five days after the elicitation, the plants were collected and used in the further analysis. A portion of the samples were freeze-dried. The experiments were conducted in triplicate. 

### 4.2. Qualitative-Quantitative Analysis of Lovage Phenolic Compounds Using the UPLC-MS Technique 

Methanolic extracts were prepared (0.25 g dw in 10 ml of 50% methanol, sonication in 30 °C for 1 h, and then centrifugation at 9000× *g* for 30 min) for determination and identification of phenolic compounds in the lovage leaves.

The analyses were carried out at the Institute of Soil Science and Plant Cultivation (Puławy, Poland). Ultra-Performance Liquid Chromatography-Mass Spectrometry (UPLC-MS) analyses of polyphenol extracts were performed using Waters Acquity UPLC™ system (Waters Corp., Manchester, UK) equipped with a binary pump system, a thermostatted sample manager, a thermostatic column oven, a photo diode array (PDA) detector, and a triple quadrupole detector (TQD). All analyses were carried out with the following mobile phases: mobile phase A (0.1% formic acid in Milli-Q water, *v*/*v* - Merck Lichropur Formic acid 98–100% for LC-MS), mobile phase B (0.1% formic acid in acetonitrile, *v*/*v* - Merck Lichrosolv Acetonitrile hypergrade for LC-MS). The analyses were divided into three groups:

I - quantitative analysis of LC-MS/MS of free phenolic acids [identification based on comparison with standards] - multiple reaction monitoring was used. The following instrumental parameters were applied [negative ionization mode]: capillary voltage 3.0 kV; source temperature 140 °C and desolvation temperature 350 °C; drying gas 800 L/h; cone gas 100 L/h, and collision gas, 6 mL/h. Chromatographic separation was done on a Waters Acquity UPLC HSS-C18 column, 2.1 × 100 mm, 1.8 μm (Waters Corp., Manchester, UK) equipped with a pre-column Acquity UPLC HSS-C18 VanGuard, 2.1 × 5mm, 1.8 μm (Waters Corp., Manchester, UK) at a temperature of 30 °C and a flow speed of 0.5 mL min^−1^ in the following gradient program: 0–0.5 min, 8% B; 0.5–8.0 min, 20% B; 8.1–10.0 min, 95% B; 10.1–12.0 min, 8% B. The method was validated previously by Czaban et al. [40].

II - quantitative analysis of phenolic acid conjugates - identification based on a comparison with the chlorogenic acid standard and UV absorption spectra and in-source collision induced dissociation (isCID-MS) spectra for the other compounds. Chlorogenic acid (5-caffeoylquinic acid, retention time —3.73 min, λ_max_—218, 325 nm) was used as a group standard during detection at 320 nm. MS analyses were carried out with negative and positive ionization. The following instrumental parameters were applied: capillary voltage, 2.8 kV(−)/3.1 kV (+); source temperature 140 °C, and desolvation temperature 350 °C; drying gas 800 L/h; cone gas 100 L/h, and collision gas 6 mL/h. The scanning range for both polarizations was 120–1800 *m*/*z*. The PDA detection included a range of 195–495 nm, a scanning frequency of 10 Hz, with a resolution of 3.6 nm. Chromatographic separation was done at a temperature of 55 °C and a flow speed of 0.6 mL min^−1^ on a Waters Acquity UPLC HSS T3, 2.1 × 100 mm, 1.8 μm column equipped with a pre-column Acquity UPLC HSS T3 VanGuard, 2.1 × 5 mm, 1.8 μm, and in the following gradient program: 0–26.9 min, 5% B; 26.9–27.0 min, 80% B; 27.0–28.5 min, 99% B; 28.6–30.0 min, 5% B.

III - quantitative analysis of flavonoid conjugates was carried out in the same analysis as the analysis of phenolic acid conjugates (identification based on UV and isCID-MS spectra). Narcissin (Isorhamnetin-3-rutinoside - retention time – 11.9 min, λ_max_ – 253, 354 nm) was used as a group standard during detection at 350 nm. The LC-MS data of individual phenolic compounds identification–Table S1.

### 4.3. Antioxidant and Anti-Inflammatory Activities

#### 4.3.1. Preparation of Extracts 

A total of 0.5 g of fresh material (lovage leaves) was ground with a mortar and pestle with 5 mL of solvent ethanol:water:HCl (70:29:1 *v/v*/*v*). Phenolic compounds were extracted for 20 min at 30 °C by sonication and then centrifuged at 9000× *g* for 30 min; this procedure was repeated two times before the supernatants were combined.

#### 4.3.2. Free Radical Scavenging Assay

Free radical scavenging activity was measured using 2,2′-azino-bis[3-ethylbenzothiazoline-6-sulphonic acid (ABTS^+●^), according to Re et al. [41], as a source of the free radicals. The antioxidant activity was related to Trolox (an analogue of vitamin E) and expressed as μmol of Trolox per gram of fresh weight (FW) (TEAC, Trolox equivalent antioxidant activity). The standard curve was prepared in a Trolox concentration range of 0–1500 μmol (r^2^ = 0.978).

#### 4.3.3. Ferric Reducing Antioxidant Power

Ferric reducing antioxidant power (RP) was determined according to the methods described by Oyaizu [42]. Reducing power was expressed as a Trolox equivalent (TE) in µmol of Trolox per gram of fresh weight (FW).

#### 4.3.4. Chelating Power

Chelating power (CHP) was determined using the method developed by Guo et al. [43]. The percentage of inhibition of ferrozine-Fe^2+^ complex formation was calculated using the formula:

% inhibition = [1 – A_A_/A_C_] × 100; where: 

A_C_—absorbance of the control (the solvent instead of the extract), A_A_—absorbance of the sample.

Chelating power was expressed as an EDTA equivalent in µg EDTA per g of fresh weight (FW). The standard curve was prepared in the EDTA concentration range of 0–15 μg mL^−1^ (r^2^ = 0.996).

### 4.4. Lipoxygenase and Cyclooxygenase Inhibitory Activity 

The ability to inhibit lipoxygenase (LOXI) activity was determined according to Szymanowska et al. [44] adapted to a microplate reader. One unit of LOX activity was defined as an increase in absorbance of 0.001 per minute at 234 nm. The changes in absorbance at 234 nm were measured using a BioTek Microplate Reader. The corresponding control contained the same concentration of the enzyme with the absence of the inhibitor. An extract concentration (mg FW/mL) providing 50% inhibition (EC50) was obtained by plotting the inhibition percentage against the sample concentrations. All assays were measured in triplicate.

A Cayman Chemical COX Colorimetric Inhibitor Screening Assay Kit was used to determine the level of cyclooxygenase 2 (COX 2) inhibition. An extract concentration (mg FW ml^−1^) providing 50% inhibition (EC50) was obtained by plotting the inhibition percentage against the sample concentrations.

### 4.5. Statistical Analysis

All results were means of three independent experiments. The presented data show mean values ± standard deviation (*n* = 9). The results were evaluated for statistical significance using univariate analysis of variance (ANOVA) with Statistica 6.0 software (StatSoft, Inc., Tulsa, OK, USA) and Tukey’s post hoc test, where the elicitor type was used as a factor. Differences were considered significant at *p* < 0.05.

## 5. Conclusions

Based on the results demonstrating the particular elicitation-induced increase in the phenolic compound content and biological activity of lovage leaves, referred to as antioxidant and potentially anti-inflammatory properties, 0.1% yeast extract (YE2) and 10 µM jasmonic acid (JA2) proved to be the most effective concentrations of the elicitors.

## Figures and Tables

**Table 1:** Qualitative and quantitative analysis of phenolics in the control and elicited lovage.

No.	Compound [mg/g dw]	Sample						
		C	JA1	JA2	JA3	YE1	YE2	YE3
1	4-caffeoylquinic acid	4.04 ± 0.46 ^a,b,c^	4.44 ± 0.12 ^b,c^	6.88 ± 0.67 ^d^	5.51 ± 0.47 ^c,d^	3.19 ± 0.15 ^a,b^	2.82 ± 0.59 ^a,b^	2.28 ± 0.52 ^a^
2	5-caffeoylquinic acid	10.09 ± 2.92 ^a^	9.39 ± 0.16 ^a^	19.12 ± 4.11 ^b^	13.92 ± 1.12 ^a,b^	7.21 ± 0.30 ^a^	9.41 ± 2.33 ^a^	6.01 ± 1.59 ^a^
3	Caffeoylquinic acid (unknown isomer)	1.24 ± 0.32 ^a,b^	1.04 ± 0.01 ^a^	2.04 ± 0.41 ^b^	1.99 ± 0.20 ^b^	1.07 ± 0.03 ^a^	0.94 ± 0.17 ^a^	0.91 ± 0.17 ^a^
4	Quercetin 3-*O*- deoxyhexoside-*O*- hexoside	0.88 ± 0.27 ^a^	0.66 ± 0.01 ^a^	1.05 ± 0.26 ^a^	0.86 ± 0.12 ^a^	0.76 ± 0.06 ^a^	0.94 ± 0.24 ^a^	0.51 ± 0.13 ^a^
5	Apterin	10.73 ± 0.20 ^a^	10.38 ± 0.21 ^a^	13.94 ± 2.67 ^a^	10.95 ± 0.86 ^a^	9.44 ± 0.60 ^a^	10.42 ± 2.46 ^a^	9.38 ± 2.21 ^a^
6	Rutin	13.80 ± 3.99 ^a^	12.01 ± 0.10 ^a^	18.60 ± 4.16 ^a^	14.37 ± 1.26 ^a^	12.16 ± 0.86 ^a^	13.53 ± 3.38 a	9.85 ± 2.62 ^a^
7	Kemferol 3-*O*- deoxyhexoside-*O*- hexoside	1.29 ± 0.33 ^a^	1.43 ± 0.04 ^a^	1.65 ± 0.34 ^a^	1.31 ± 0.11 ^a^	1.38 ± 0.11 ^a^	1.81 ± 0.43 ^a^	1.05 ± 0.28 ^a^
8	Caffeic acid and apterin ester	1.86 ± 0.06 ^a,b^	1.12 ± 0.04 ^a^	2.99 ± 0.58 ^c^	2.45 ± 0.18 ^b,c^	1.59 ± 0.06 ^a,b^	1.28 ± 0.20 ^a^	1.26 ± 0.24 ^a^
9	Sinapic acid and apterin ester [5]	1.32 ± 0.28 ^a,b^	1.00 ± 0.01 ^a^	2.08 ± 0.12 ^c^	1.69 ± 0.12 ^b,c^	1.30 ± 0.06 ^a,b^	1.38 ± 0.24 ^a,b^	1.10 ± 0.18 ^a,b^
10	p-coumaric acid and apterin ester	0.59 ± 0.06 ^a,b^	0.44 ± 0.01 ^a^	0.75 ± 0.12 ^b^	0.63 ± 0.03 ^a,b^	0.56 ± 0.02 ^a,b^	0.61 ± 0.07 ^a,b^	0.50 ± 0.03 ^a^
11	Ferulic acid and apterin ester	0.84 ± 0.14 ^a,b^	0.62 ± 0.01 ^a^	1.03 ± 0.16 ^b^	0.93 ± 0.06 ^a,b^	0.86 ± 0.05 ^a,b^	0.86 ± 0.12 ^a,b^	0.76 ± 0.10 ^a,b^
12	(E/Z)-Ligustilide [6]	0.93 ± 0.10 ^a^	1.54 ± 0.06 ^b^	2.73 ± 0.06 ^d^	2.03 ± 0.14 ^c^	1.59 ± 0.05 ^b^	1.63 ± 0.04 ^b,c^	1.49 ± 0.21 ^b^
13	(E/Z)-Ligustlide	0.89 ± 0.31 ^a^	1.57 ± 0.11 ^a,b^	2.38 ± 0.31 ^b^	1.97 ± 0.08 ^b^	1.71 ± 0.01 ^a,b^	2.40 ± 0.28 ^b^	1.85 ± 0.17 ^b^
14	Caffeic acid [µg/g dw]	5.28 ± 0.08 ^b^	nd	6.27 ± 0.24 ^c^	14.42 ± 0.11 ^d^	nd	nd	2.63 ± 0.78 ^a^
Sum		48.43 ± 4.02 ^a^	45.63 ± 0.77 ^a^	75.25 ± 8.68 ^b^	58.63 ± 4.74 ^a,b^	42.81 ± 2.36 ^a^	48.02 ± 9.99 ^a^	36.96 ± 8.45 ^a^

Abbreviations: C–control plants, JA1–plants elicited with 1 µM jasmonic acid, JA2–plants elicited with 10 µM jasmonic acid, JA3–plants elicited with 100 µM jasmonic acid; YE1–plants elicited with 0.01% yeast extract, YE2–plants elicited with 0.1% yeast extract, YE3–plants elicited with 1% yeast extract. Means (± SD) in rows followed by different letters are statistically significantly different at *p* < 0.05. nd–not detected.

## Supplementary Materials

The following are available online. Table S1: LC-MS data and individual phenolic compounds identification in lovage.
