# Supplementary material for: Antioxidative and Potentially Anti-inflammatory Activity of Phenolics from Lovage Leaves Levisticum officinale Koch Elicited with Jasmonic Acid and Yeast Extract
Source: Molecules. 2019 Apr 11;24(7):1441. doi: 10.3390/molecules24071441 (PMC6480578; doi:10.3390/molecules24071441)
Supplement: Supplementary file 1 [file molecules-24-01441-s001.pdf]

molecules

MDPI

Supplementary material

Antioxidative and anti-inflammatory activity of phenolics from lovage leaves [*Levisticum officinale* Koch] elicited with jasmonic acid and yeast extract

Urszula Złotek, Urszula Szymanowska, Łukasz Pecio, Solomiia Kozachok and Anna Jakubczyk

Table S1. LC-MS data and individual phenolic compounds identification in lovage

Compound	Rt [min]	λ_{max} [nm]	MS *m/z* $[M-H]^-$ / $[M+H]^+$	MS/MS *m/z* $[M-H]^-$ / $[M+H]^+$
4-caffeoylquinic acid	1.96	211; 219; 322	353 / 355	191; 179 / 163
5-caffeoylquinic acid	3.73	217; 324	353 / 355	191 / 163
Caffeoylquinic acid (unknown isomer)	4.28	217; 325	353 / 355	191; 179; 173 / 163
Quercetin 3-O- deoxyhexoside-O-hexoside	9.93	253; 356	609 / 611	465; 303
Apterin	10.18	263; 336	423 / 425	243 / 263; 245
Rutin	10.25	255; 354	609 / 611	465; 303
Kemferol 3-O- deoxyhexoside-O-hexoside	11.44	265; 332	593 / 595	449; 287
Caffeic acid and apterin ester	13.02	265; 330	585 / 587	325; 263; 181; 163
Sinapic acid and apterin ester [5]	13.95	264; 330	629 / 631	369; 351; 263; 207
p-coumaric acid and apterin ester	14.19	266; 318	569 / 571	309; 263; 147
Ferulic acid and apterin ester	14.33	265; 330	599 / 601	339; 321; 263; 177
(E/Z)- Ligustilide [6]	20.19	222; 292; 327	191	173; 163; 145
(E/Z)- Ligustilide	20.78	222; 279; 328	191	173; 163; 145
